# Stimulation of Non-canonical NF-κB Through Lymphotoxin-β-Receptor Impairs Myogenic Differentiation and Regeneration of Skeletal Muscle

**DOI:** 10.3389/fcell.2021.721543

**Published:** 2021-10-05

**Authors:** Manuel Schmidt, Anja Weidemann, Christine Poser, Anne Bigot, Julia von Maltzahn

**Affiliations:** ^1^Leibniz Institute on Aging, Fritz Lipmann Institute, Jena, Germany; ^2^Center of Research in Myology-UMRS 974, Institute of Myology, INSERM, Sorbonne Université, Paris, France

**Keywords:** NF-kappa B, NF-κB, LTβR, lymphotoxin-β-receptor, muscle stem cell, satellite cell, regeneration, myogenic differentiation

## Abstract

Myogenic differentiation, muscle stem cell functionality, and regeneration of skeletal muscle are cellular processes under tight control of various signaling pathways. Here, we investigated the role of non-canonical NF-κB signaling in myogenic differentiation, muscle stem cell functionality, and regeneration of skeletal muscle. We stimulated non-canonical NF-κB signaling with an agonistically acting antibody of the lymphotoxin beta receptor (LTβR). Interestingly, we found that stimulation of non-canonical NF-κB signaling through the LTβR agonist impairs myogenic differentiation, muscle stem cell function, and regeneration of skeletal muscle. Furthermore, we show that stimulation of non-canonical NF-κB signaling by the LTβR agonist coincides with activation of canonical NF-κB signaling. We suggest a direct crosstalk between canonical and non-canonical NF-κB signaling during myogenic differentiation which is required for proper myogenic differentiation and thereby regeneration of skeletal muscle.

## Introduction

Skeletal muscle makes up approximately 40% of the total body weight of an adult, it contributes to several bodily functions such as voluntary movements and breathing. Furthermore, skeletal muscle can adapt to physiological demands such as growth, training, or injury (Frontera and Ochala, 2015). It also has a remarkable ability to regenerate, a process which depends on muscle stem cells (MuSCs), also termed satellite cells (Lepper et al., 2011; Murphy et al., 2011; Brack and Rando, 2012; Wang and Rudnicki, 2012; Wang et al., 2014). MuSCs are characterized by the expression of the transcription factor Pax7 which is essential for their functionality (Lepper et al., 2009; von Maltzahn et al., 2013). Under resting conditions, they are quiescent and located between the plasmalemma of the myofiber and the basement membrane (Wang et al., 2014; Frontera and Ochala, 2015; Ancel et al., 2021). Upon injury, MuSCs become activated, proliferate, differentiate, and replace damaged muscle tissue (Schmidt et al., 2019). One remarkable characteristic of MuSCs is their ability to self-renew thereby sustaining the stem cell pool over multiple cycles of growth and regeneration (Brack and Rando, 2012; Wang and Rudnicki, 2012; Wang et al., 2014). The functionality of MuSCs is a tightly controlled process, intrinsic as well as extrinsic cues affect MuSC proliferation, differentiation, and self-renewal. The extrinsic signals can derive from the stem cell niche or from the blood stream (systemic factors) (Bentzinger et al., 2010; Chang and Rudnicki, 2014; Dinulovic et al., 2017). Intrinsic changes in MuSCs or in their direct niche can dramatically compromise MuSC functionality resulting in impaired regeneration of skeletal muscle as observed during aging (Price et al., 2014; Sousa-Victor et al., 2014; Brack and Munoz-Canoves, 2016; Schwörer et al., 2016).

Regeneration of skeletal muscle resembles embryonic myogenesis, differentiation of MuSCs and myogenic progenitor cells is accompanied by a transcription factor cascade comprising key myogenic regulatory factors including MyoD, myf5, myogenin, and myogenic regulatory factor 4 (MRF-4) (Chal and Pourquie, 2017; Hernandez-Hernandez et al., 2017). Activation of MuSCs coincides with the upregulation of MyoD, a transcription factor promoting myogenic differentiation (Hernandez-Hernandez et al., 2017; Ancel et al., 2021). Activated/committed MuSCs then progress in the myogenic program to become myogenic progenitor cells which are characterized by the expression of MyoD while expression of Pax7 is lost. Upon induction of myogenin expression, the cells further differentiate to become myocytes which then fuse to form multinucleated myotubes. In the final step of myogenesis, those myotubes mature into myofibers, which are the functional units of skeletal muscle (Forcina et al., 2019; Schmidt et al., 2019).

Nuclear factor-κB (NF-κB) signaling plays an important role during differentiation in multiple tissues including skeletal muscle (Ghosh and Karin, 2002; Hayden and Ghosh, 2004; Kucharczak et al., 2004). The NF-κB family comprises five members: p65/RelA, RelB, c-Rel, p100/p52, and p105/p50, which can form homo- or heterodimers. Depending on the NF-κB signaling molecules, one can divide NF-κB signaling into the canonical (classical) and non-canonical (alternative) pathway (Oeckinghaus and Ghosh, 2009; Zhang et al., 2017). Activation of the canonical pathway by TNF-α leads to the phosphorylation of inhibitor of kappa B (IκB), which causes the sequestration of p65/RelA and p50 into the cytoplasm. Hence, NF-κB transcription factors (like p65/RelA, p50, and c-Rel) shuttle into the nucleus and initiate expression of target genes which then drive proliferation, differentiation, or survival pathways (Shih et al., 2011). While the canonical NF-κB pathway is activated by TNF-α, the non-canonical pathway can be stimulated by binding of lymphotoxin α and β to lymphotoxin-β-receptor (LTβR). After activation of the non-canonical NF-κB pathway, the precursor protein p100 is phosphorylated by a IκB kinase α (IKKα) homodimer, which mediates the partial proteolysis of p100 thereby generating the active form p52. p52 then forms a heterodimer with RelB and translocates into the nucleus (Oeckinghaus and Ghosh, 2009; Zhang et al., 2017). Of note, non-canonical NF-κB signaling was demonstrated to be independent of IKKβ (Mackay et al., 1996; Muller and Siebenlist, 2003; Shih et al., 2011).

In recent years the role of NF-κB signaling during myogenesis has been investigated focusing on the impact of the canonical pathway. Canonical NF-κB signaling is thought to control myogenesis by limiting myogenic differentiation. For instance, deletion of p65/RelA was shown to enhance the expression of myogenic transcription factors thereby accelerating myogenic differentiation (Bakkar et al., 2008). While myogenic differentiation was thought to be mainly controlled by canonical NF-κB signaling, non-canonical NF-κB signaling was demonstrated to be important for myotube maintenance and metabolic regulation. For instance, MyoD in cooperation with RelB regulates the oxidative metabolism through binding to enhancer sites of the PGC-1β gene thereby regulating mitochondrial biogenesis (Bakkar et al., 2012; Shintaku et al., 2016).

Here, we investigated how stimulation of non-canonical NF-κB signaling affects myogenic differentiation, muscle stem cell function, and regeneration of skeletal muscle. We demonstrate that stimulation of the non-canonical NF-κB signaling pathway via the LTβR impairs myogenic differentiation and limits muscle stem cell functionality. This then leads to impaired regeneration of skeletal muscle. Interestingly, we observe impaired regeneration and myogenic differentiation following stimulation of non-canonical NF-κB signaling in wt and in mdx mice. Furthermore, we noticed a compensatory upregulation of members of the canonical NF-κB pathway following stimulation of the LTβR. This suggests that a tight regulation and crosstalk of canonical and non-canonical NF-κB signaling is essential for proper myogenic differentiation and muscle stem cell functionality.

## Materials and Methods

### Mice

Adult (12 weeks of age) male C57/BL6JRj mice obtained from Janvier Laboratories or male mdx mice (12 weeks of age) from Charles River Laboratories were used in this study. All mice were maintained inside a SPF facility. All animal procedures were in accordance with the European Union (EU) directive 2010/63/EU and approved by the Animal Welfare Committee of the Thüringer Landesamt für Lebensmittelsicherheit und Verbraucherschutz (TLV; 03-011/14; Bad Langensalza, Germany).

For regeneration experiments, TA muscles of WT or mdx mice were injured by intramuscular injection of 50 μl CTX (10 μM) followed by injection of solvent (0.9% NaCl), LTβR agonist (10 μg for WT and 30 μg for mdx mice) or LTβR antagonist (30 μg) at 3 days post injury (dpi) under isoflurane anesthesia. Muscles were isolated 10 or 14 dpi, embedded in cryoprotective medium (Tissue-Tek O.C.T. with 10% sucrose) and frozen in liquid nitrogen.

### Cell Culture, Transfection and Modulation of NF-κB Signaling

Muscle stem cells were isolated via FACS from hind-limb muscles of 12-week-old male C57/BL6JRj with a positive selection for α-7-Integrin as described before (Pasut et al., 2012), cultured and plated on collagen-coated dishes (Corning) in culture medium containing Ham’s F10 medium (Thermo Fisher Scientific), 20% fetal bovine serum (FBS) (Thermo Fisher Scientific), 2% Penicillin/Streptomycin (Thermo Fisher Scientific), and 5 ng/ml basic fibroblast growth factor (bFGF) (Thermo Fisher Scientific). Differentiation of primary murine myoblasts was initiated by a switch of culture media to differentiation medium which contains DMEM (Thermo Fisher Scientific), 10% HS (horse serum, Thermo Fisher Scientific), and 2% Penicillin/Streptomycin (Thermo Fisher Scientific). Human myoblasts from DMD patients and healthy controls [AB1190 Clone 1 – healthy, AB1023DMD11Q Clone 1 and AB1071DMD13PV Clone 5 – DMD, were cultured in skeletal muscle cell growth media (PromoCell, C-23060) with 15% FBS (Thermo Fisher Scientific), 1% GlutaMAX (Thermo Fisher Scientific), 1% Gentamicine (Thermo Fisher Scientific), and 1% Penicillin/Streptomycin (Thermo Fisher Scientific)]. Differentiation of human myoblasts was initiated with skeletal muscle differentiation medium (PromoCell, C-23061) and 1% Penicillin/Streptomycin (Thermo Fisher Scientific). Murine or human myoblasts and MuSCs on floating myofibers were transfected with either non-targeting siRNA (Dharmacon) or si-Rela (Dharmacon) with Lipofectamine RNAiMAX (Thermo Fisher Scientific) according to the protocol provided by the manufacturer. Stimulation or inhibition of non-canonical NF-κB signaling in murine cells was performed by addition of antibodies either acting agonistically (LTβR agonist; Aldevron; concentration: 1 μg/μl) or acting antagonistically (LTβR antagonist; concentration: 0.6 μg/μl, obtained from T. Hehlgans, Regensburg, Germany) to the differentiation medium (Weidemann et al., 2016). As a control a mouse IgG antibody (Sigma-Aldrich, 12-370) or equal amount of the solvent (PBS) were added to the medium. Stimulation of non-canonical NF-κB signaling in human myoblasts was performed by addition of recombinant mouse anti-human LTβR antibody (CBE11) (1 μg/μl, Creative Biolabs) known to act agonistically. Canonical NFκB signaling was inhibited by addition of either TPCA-1 or IMD0454 (Abcam; 1 μM final concentration) dissolved in DMSO to the medium, as a control equal amounts of DMSO were used.

### Myofiber Isolation and Culture

Preparation and cultivation of *extensor digitorum longus* (EDL) fibers was performed as described previously (Schmidt et al., 2020). In brief, EDL muscles were dissected under sterile conditions and incubated in DMEM with 0.2% collagenase (from *Clostridium histolyticum*, Sigma-Aldrich) at 37°C in a water bath for 1 h. Dissociation of single myofibers was accomplished by trituration with sterile glass pipettes and single myofibers were cultured in myofiber culture medium (DMEM, 20% FBS, 1% chicken embryo extract; United States Biological) at 37°C, 5% CO_2_ and 95% humidity for 72 h. The LTβR agonist (concentration: 1 μg/μl) or LTβR antagonist (concentration: 0.6 μg/μl) were added to the culture medium directly after isolation. Myofibers with their adjacent muscle stem cells were fixed with 2% paraformaldehyde (PFA) for 5 min, washed twice with PBS (pH 7.4) and permeabilized with PBS, 0.1% Triton X-100, 0.1 M glycine (pH 7.4) for 10 min. Myofibers were then incubated with blocking solution [5% HS in PBS (pH 7.4)] for 1 h followed by incubation with the primary antibodies directed against Pax7 (undiluted, PAX7; DSHB) and MyoD (1:100, Invitrogen, #PA5-23078) at 4°C overnight. After three washing steps with PBS (pH 7.4) the myofibers were incubated with the secondary antibodies for 45 min at room temperature (RT). Afterwards, myofibers were washed three times with PBS (pH 7.4), incubated with DAPI (50 μg/ml) for 5 min, washed twice with PBS (pH 7.4) and then mounted in PermaFluor mounting medium (Thermo Fisher Scientific) on glass microscope slides. Analysis was carried out using an Axio Observer D1 (Carl Zeiss).

### Histochemistry

Muscles frozen in liquid nitrogen were cut in 14 μm cross-sections. Hematoxylin-Eosin (H&E) staining was performed as described earlier (von Maltzahn et al., 2013) with the Leica stainer XL. Images were taken with the Axio Observer D1 (Carl Zeiss).

### Immunostaining and Antibodies

Muscles frozen in liquid nitrogen were cut in 14 μm cross-sections. Cross-sections were fixed with 2% PFA for 10 min, washed twice with PBS (pH 7.4), permeabilized with 0.1% Triton X-100, 0.1 M glycine in PBS (pH 7.4) for 10 min, incubated first with blocking solution [5% HS in PBS (pH 7.4)] and then with primary antibodies in blocking solution at 4°C overnight. Afterwards, samples were washed three times with PBS (pH 7.4) and incubated with secondary antibodies in blocking solution for 45 min at RT. After washing with PBS (pH 7.4) three times, nuclei were counterstained with DAPI (50 μg/ml) and washed twice with PBS (pH 7.4). Cross-sections were mounted in PermaFluor (Thermo Fisher Scientific). The following antibodies were used: mouse anti-myosin (undiluted, MF20; DSHB), mouse anti-myogenin (undiluted, F5D, DSHB), mouse anti-Pax7 (undiluted, PAX7, DSHB), rabbit anti-MyoD (1:250, Thermo Fisher Scientific, #PA5-23078), chicken anti-Laminin (1:500, LC Bio, LS-C96142), rabbit anti-Laminin (1:1000, MERCK, L9393), and mouse anti-embryonic MHC (undiluted, F1.652, DSHB). Analyses were carried out using the Axio Observer D1 or the Axiovert 200 Apotome (Carl Zeiss). The number of Pax7^+^ MuSCs was enumerated by counting Pax7^+^ cells per muscle cross-section divided by the total area of the cross-section.

### Immunoblot Analyses

Mouse and human myoblasts were lysed in RIPA buffer for 20 min on ice followed by sonication (10 cycles, 30 s on and 5 s off). Between 10 and 20 μg of protein were separated on a 8% Bis-Tris protein gel. Then, a transfer to a nitrocellulose membrane (VWR) was carried out followed by incubation of the membrane in blocking solution (3% BSA in TBST) for 1 h at RT and then with primary antibodies in blocking solution at 4°C overnight. Afterward, membranes were washed three times with TBST, incubated with secondary antibodies in blocking solution for 45 min at RT and washed three times with TBST. Membranes were incubated with Pierce^TM^ ECL Western Blotting Substrate (Thermo Fisher Scientific) and imaged using the Bio-Rad MyImager. Cytoplasmatic and nuclear fractions were generated as described previously (Weidemann et al., 2016). The following primary antibodies were used: rabbit anti-RelA (1:200, Santa Cruz, sc-372), rabbit anti-phospho-RelA (1:1000, Cell Signaling, #3033), rabbit anti- NF-κB2 p100/p52 (1:1000, Cell Signaling, #4882), rabbit anti-RelB (1:1000, Cell Signaling, #4882), rabbit anti-phospho RelB (Ser552) (1:1000, Cell Signaling, #4999S), rabbit anti-IKBα (1:1000, Cell Signaling, #2859), rabbit anti-phospho-IKKα/β (1:1000, Cell Signaling, #2697), mouse anti-myosin (undiluted, MF20, DSHB), mouse anti-myogenin (undiluted, F5D, DSHB), rabbit anti-MyoD (1:100, Thermo Fisher Scientific, #PA5-23078), rabbit anti-LTβR (1:500, Abcam, ab70063), mouse anti-GAPDH (1:200, Santa Cruz, sc-365062), mouse anti-PARP-1 (C2-10) (1:4000, Santa Cruz, sc-53643). The following secondary antibodies were used: goat anti-rabbit Immunoglobulins/HRP (1:1000, Dako, P0448), goat anti-mouse Immunoglobulins/HRP (1:1500, Dako, P0447).

### Real-Time PCR

Total RNA was isolated with TriFast (PeqLab) reagent followed by additional purification using the Qiagen RNA isolation kit. Reverse transcription was performed with the iScript cDNA synthesis kit (Bio-Rad) according to the protocol provided by the manufacturer. SYBR Green real-time PCR (IQ SYBR Supermix, Bio-Rad) was performed on a real-time thermocycler (Bio-Rad). Primers used are listed in Table 1.

**TABLE 1:** Primers used for quantitative RT-PCR.

Name	Forward sequence	Reverse sequence
MyoD	CTA CAG TGG CGA CTC AGA T	CAC TGT AGT AGG CGG TGT C
Myogenin	CAG TAC ATT GAG CGC CTA C	AAG GCA ACA GAC ATA TCC TC
Mef2a	GAT GCT GAC GAT TAC TTT GA	TCG AAT CTG CTA ATG TTG AG
PGC1α	GAG TCT GAA AGG GCC AAA CA	ACG GTG CAT TCC TCA ATT TC
LTβR	TTA TCG CAT AGA AAA CCA GAC TTG C	TCA AAG CCC AGC ACA ATG TC
NF-κB2 p100	GCT AAT GTG AAT GCC CGG AC	CTT TGG GTA TCC CTC TCA GGC
Relb	GTG ACC TCT CTT CCC TGT CAC TAA C	CAG CGT GAC GCT GCT CAG T
Rela	CCT CTC ACA TCC GAT TTT TGA T	GTC CCG TGA AAT ACA CCT CAA T
GAPDH	ATG CCA GTG AGC TTC CCG TC	CAT CAC CAT CTT CCA GGA GC

### Statistical Analysis

A minimum of three and a maximum of five replicates were analyzed for each experiment presented. Data are shown as SEM. Statistical significance was assessed by Student’s *t* test (unpaired, two-tailed) or one-way or two-way ANOVA, using Microsoft Excel or Prism after testing for normal distribution using the Shapiro–Wilk test. In cases where the Shapiro–Wilk test failed, a Kruskal–Wallis test was used (indicated in the respective figure legend). A *p* value < 0.05 was considered significant.

## Results

### Activation of Non-canonical NF-κB Signaling by Stimulating the LTβR Impairs Myogenic Differentiation

**FIGURE 1:**
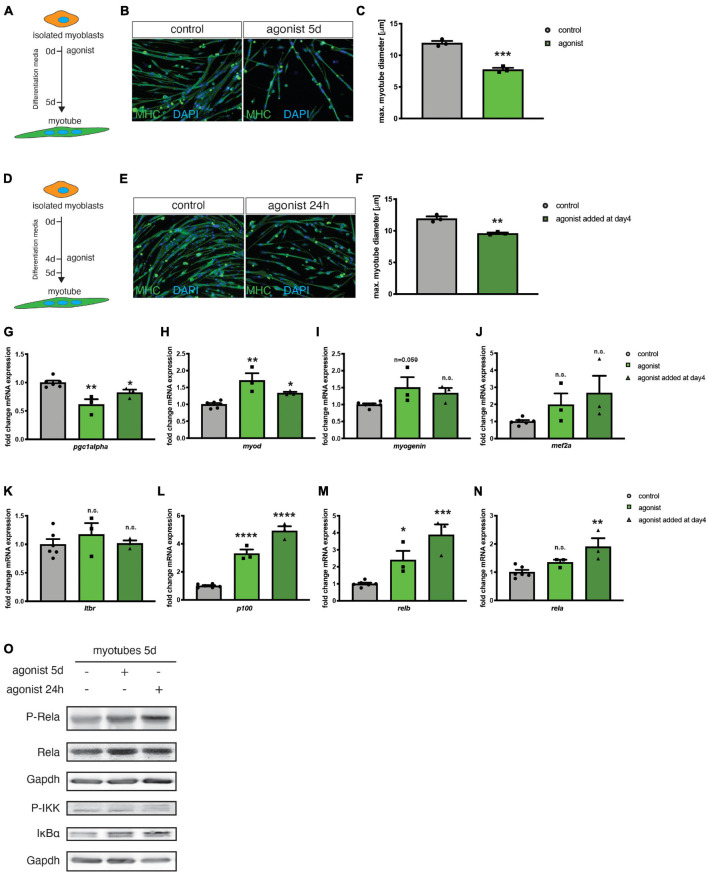
Activation of the LTβR pathway impairs myogenic differentiation. **(A)** Experimental scheme of myoblast differentiation in the presence of the LTβR agonist. **(B)** Representative images of myotubes incubated with the control or the LTβR agonist with antibodies directed to myosin heavy chain (MHC, green). Nuclei are counterstained with DAPI (blue). Scale bar = 50 μm. **(C)** 5 days culture of myoblasts in the presence of the LTβR agonist markedly reduces myotube diameter compared to control treatments. *n* = 3. **(D)** Experimental scheme of myoblast differentiation with incubation of myotubes at day 4 of differentiation for 24 h with the LTβR agonist or a control. **(E)** Representative images of myotubes incubated with the control or the LTβR agonist with antibodies directed to myosin heavy chain (MHC, green). Nuclei are counterstained with DAPI (blue). Scale bar = 50 μm. **(F)** Incubation at day 4 of differentiation of 24 h with the LTβR agonist results in reduced myotube diameter. *n* = 3. **(G)** qRT-PCR for *pgc1*α. qRT-PCR of myotubes after incubation with the LTβR demonstrates significant upregulation of m*yoD* **(H)** and *myogenin* **(I)**, but not *mef2a* **(J)**. qRT-PCR analysis of LTβR pathway target genes shows no markedly influence on LTβR expression **(K)**, but a significantly increase of p100 **(L)**, *relb* **(M)** and classical NF-κB target gene *rela* mRNA **(N)**, especially after incubation at day 4 for 24 h with the LTβR agonist. *n* = 3. **(O)** Activation of the canonical NF-κB pathway by the LTβR agonist shown by increased p-RelA and p-IKK. *p < 0.05, **p < 0.01, ***p < 0.001, ****p < 0.0001. Error bars represent SEM. In **(N)** a Kruskal–Wallis test was used.

So far, mainly the role of canonical NF-κB signaling during myogenic differentiation and MuSC function has been investigated in detail (Bakkar et al., 2008; Bakkar and Guttridge, 2010; He et al., 2013). The role of non-canonical NF-κB signaling during myogenic differentiation, especially its activation via the LTβR remained elusive. To determine the activation status of non-canonical NF-κB signaling during myogenic differentiation, we first investigated expression and phosphorylation (corresponding to the activation status) of members of the canonical and non-canonical NF-κB signaling pathway during myogenic differentiation in primary murine myoblasts. Interestingly, we found that members of both, the canonical and the non-canonical signaling pathway are activated during early myogenic differentiation. For instance, we detected an increase in the phosphorylated/activated forms of RelA and RelB during myogenic differentiation suggesting that also non-canonical NF-κB signaling plays an important role during myogenic differentiation (Supplementary Figures 1A,B). To investigate the role of non-canonical NF-κB signaling in myogenic differentiation, we differentiated primary murine myoblasts for 5 days in the presence of an antibody directed against the LTβR (LTβR agonist) known to stimulate non-canonical NF-κB signaling through binding to the LTβR (Figure 1A and Supplementary Figures 1C,D; Lukashev et al., 2006; Weidemann et al., 2016). Stimulation of non-canonical NF-κB signaling at induction of differentiation caused a significant decrease in myotube size compared to myotubes which were differentiated in the presence of the solvent or an IgG control antibody (Figures 1B,C). Next, we asked whether stimulation of non-canonical NF-κB signaling exclusively affects early myogenic differentiation or if myotube maintenance is also affected. Therefore, we differentiated myoblasts for 4 days to obtain multinucleated myotubes and then added the LTβR agonist followed by an additional day of culture in differentiation medium (Figure 1D). Interestingly, also this stimulation for 24 h of already formed myotubes resulted in a reduction of myotube diameter suggesting that also myotube maintenance is affected or that activation of non-canonical NF-κB signaling can cause myotube atrophy. However, the effect size was reduced compared to activation of non-canonical NF-κB signaling at the induction of myogenic differentiation (Figures 1E,F). We further confirmed that addition of the LTβR agonist activates non-canonical NF-κB signaling in myogenic cells. Stimulation with the LTβR agonist results in the cleavage of the precursor p100 into the active form p52 in myotubes which were differentiated in the presence of the LTβR agonist (Supplementary Figures 1C,D). Surprisingly, we also detected an increased phosphorylation of RelA, a member of the canonical NF-κB pathway in both conditions (Supplementary Figures 1C,D and Figure 1O). Hence, we conclude that stimulation of non-canonical NF-κB signaling with the LTβR agonist impairs myogenic differentiation and myotube maintenance while also resulting in the activation of the canonical NF-κB pathway.

Non-canonical NF-κB was reported to be involved in myotube maintenance and metabolic regulation involving *pgc1*α (Bakkar et al., 2012; Shintaku et al., 2016). Therefore, we investigated the expression of the mitochondrial biogenesis marker peroxisome proliferator-activated receptor gamma coactivator 1-alpha (*pgc1*α) after stimulating the non-canonical NF-κB pathway with the LTβR agonist. We detected a reduced expression of *pgc1*α in myotubes stimulated with the LTβR agonist (Figure 1G). Furthermore, we determined the expression of *myod* and *myogenin*, two markers of early myogenic differentiation and *mef2a*, known to control myogenic fusion. We found increased expression levels of the three markers after stimulation of the non-canonical NF-κB pathway with the LTβR agonist (Figures 1H–J). This further strengthens our finding that activation of non-canonical NF-κB signaling impairs myogenic differentiation since terminally differentiated myotubes do not express *myod* and only very few amounts of *myogenin* (Schmidt et al., 2019). *Myod* and *myogenin* are typical markers for myoblasts and myocytes, cell types which are expressed during early myogenic differentiation.

Next, we asked whether stimulation with the LTβR agonist affects mRNA expression of members of the non-canonical and canonical NF-κB signaling pathways. We found that stimulation with the LTβR agonist does not affect mRNA levels of *ltβr* (Figure 1K). However, we detected a significant increase in mRNA levels of *relb* and *p100/nfκb1 –* as expected (Figures 1L,M). We then investigated, if also the mRNA levels of members of the canonical NF-κB signaling pathway are affected. Indeed, we found mRNA levels of *rela* to be increased upon stimulation with the LTβR agonist (Figure 1N). The increased mRNA levels of *rela* coincide with increased phosphorylation of RelA and increased phosphorylation of the kinase IKKα, which inactivates the p65/RelA inhibitor IKBα (Figure 1O). Therefore, we conclude, that stimulating LTβR with the LTβR agonist results in impaired myogenesis via activation of the non-canonical NF-κB pathway concomitant with – a potentially compensatory – activation of canonical NF-κB pathway.

### Inhibiting Canonical NF-κB Signaling Counteracts Impaired Myogenic Differentiation Following Stimulation of Non-canonical NF-κB Signaling

**FIGURE 2:**
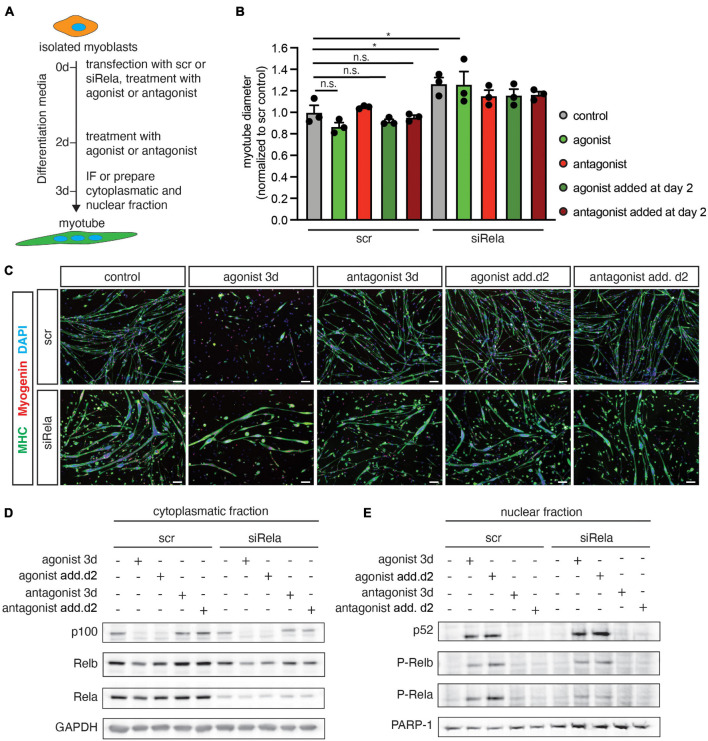
Knockdown of *rela* enhances myogenic differentiation and impairs activation of the canonical pathway by LTβR. **(A)** Experimental scheme of myoblast differentiation in the presence of the LTβR agonist or antagonist and transfection with siRNA targeting *rela* or a non-targeting control siRNA. **(B)** Incubation at the beginning of differentiation or incubation at day 3 of differentiation markedly decreases myotube diameter compared to the control. Knockdown of *rela* results in increased myotube diameters independent of incubation with the LTβR agonist or antagonist. *n* = 3. **(C)** Representative images of immunofluorescent images from myotubes quantified in **(B)**, myosin heavy chain (MHC) in green and myogenin in red. Nuclei are counterstained with DAPI (blue). Scale bar = 50 μm. **(D)** Incubation of myotubes with the LTβR agonist at the beginning of differentiation or addition at day 2 of differentiation leads to the cleavage of p100 to active p52 at day 3 of differentiation **(E)** and increased phosphorylation of RelA **(E)**. *p < 0.05. Error bars represent SEM.

Next, we asked whether active canonical NF-κB signaling is a prerequisite for impaired myogenic differentiation after stimulating the non-canonical NF-κB pathway with the LTβR agonist. Therefore, we differentiated primary murine myoblasts for 3 days in the presence of the LTβR agonist and a siRNA targeting *rela* to silence canonical NF-κB signaling (Figure 2A). As an additional control we used a LTβR antagonistic antibody to inhibit non-canonical NF-κB signaling. Stimulation of non-canonical NF-κB signaling with the LTβR agonist resulted in impaired myogenic differentiation as evidenced by a reduced myotube diameter (Figures 2B,C). This suggests that stimulation of non-canonical NF-κB signaling affects early myogenic differentiation (Figures 2B,C) as well as growth/maintenance of myotubes (Supplementary Figures 1A–F). As expected, we did not observe any effect on myogenic differentiation after inhibiting non-canonical NF-κB signaling with the LTβR antagonist (Figures 2B,C). However, knockdown of *rela* resulted in enhanced myogenic differentiation independent of stimulation or inhibition of non-canonical NF-κB signaling (Figures 2B,C). To demonstrate that indeed early myogenic differentiation is impaired through activation of non-canonical NF-κB signaling with the LTβR agonist, we analyzed the myotube diameter after 2 days of differentiation. Thereby we found the myotube size (Supplementary Figures 1E,F) to be significantly reduced when differentiation was induced in the presence of the LTβR agonist. Also inhibition of non-canonical NF-κB signaling with the LTβR antagonist in already formed myotubes did not affect their size. However, inhibition of non-canonical NF-κB signaling with TPCA-1, an inhibitor of IKKb or through silencing RelA resulted in larger myotubes independent of activation or inhibition of non-canonical NF-κB signaling with the LTβR agonist or antagonist (Supplementary Figures 1E,F).

Next, we evaluated the activation status of members of the canonical and the non-canonical NF-κB pathway after knockdown of *rela* or stimulation of the LTβR in 3 days differentiated myotubes (Figures 2D,E). Interestingly, knockdown of *rela per se* did not alter the abundance or phosphorylation status of members of the non-canonical NF-κB signaling pathway (p100, p52, RelB, p-RelB). Stimulation of non-canonical NF-κB signaling by the LTβR agonist resulted in increased p52 levels by cleavage of p100. However – in line with the results obtained earlier – we observed an increased phosphorylation of RelA after stimulation of non-canonical NF-κB signaling with the LTβR agonist. We conclude that stimulation of the LTβR impairs myogenic differentiation through activating non-canonical NF-κB signaling concomitant with activation of the canonical pathway.

### Stimulation of Non-canonical NF-κB Signaling Impairs Muscle Regeneration and Muscle Stem Cell Functionality in wt Mice

**FIGURE 3:**
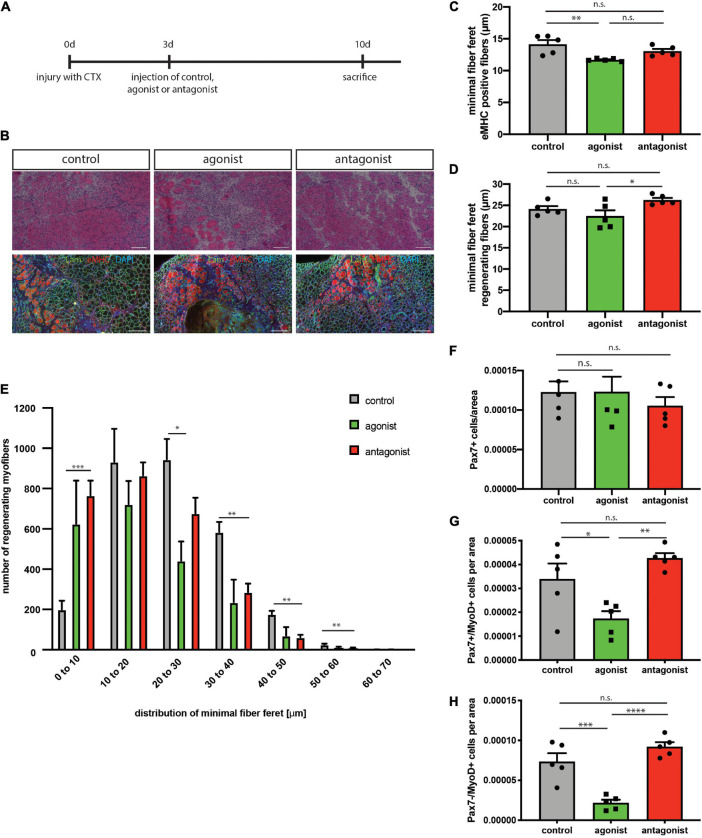
Activation of the non-canonical NF-κB signaling by the LTβR agonist impairs regeneration in wt mice. **(A)** Experimental scheme depicting CTX injury and injection of the LTβR agonist, LTβR antagonist or solvent control at day 3 after injury. **(B)** Representative images of HE staining and immunostaining with antibodies directed to laminin (green) and embryonic MHC (red). Nuclei are counterstained with DAPI (blue). Scale bar = 200 μm. **(C)** Injection of the LTβR agonist results in decreased myofiber size of eMHC-positive myofibers compared to control and antagonist injected muscles. *n* = 5. **(E)** Size distribution of myofibers undergoing regeneration as in **(D)** *n* = 5. **(F)** Number of Pax7-positive cells in regenerating muscles following injection of LTβR agonist or antagonist. *n* = 5. **(G)** Injection of the LTβR agonist reduces the number of committed MuSCs (Pax7^+^/MyoD^+^), *n* = 5. **(H)** Injection of the LTβR agonist reduces the number of myoblasts (Pax7^–^/MyoD^+^). *n* = 5. *p < 0.05, **p < 0.01, ***p < 0.001, ****p < 0.0001. Error bars represent SEM.

Regeneration of skeletal muscle depends on MuSCs and their full functionality including the ability to undergo myogenic differentiation and self-renewal. To determine, if stimulation or inhibition of non-canonical NF-κB signaling affects regeneration of skeletal muscle, we performed cardiotoxin (CTX) mediated injury of the tibialis anterior muscle of adult C57BL/6 mice. This was followed by a single injection of the solvent (control), the LTβR agonist or the LTβR antagonist into the injured muscle at day 3 after injury (Figure 3A). We found newly formed myofibers to be reduced in size [measured as minimal fiber feret, identified by staining for embryonic MHC (eMHC)] when muscles were injected with the LTβR agonist (Figures 3B,C and Supplementary Figure 2). Furthermore, all myofibers undergoing regeneration in muscles injected with the LTβR agonist displayed a smaller myofiber diameter compared to myofibers of muscles which were injected with the solvent (control) or the LTβR antagonist (Figures 3B,D,E). However, inhibition of non-canonical NF-κB signaling *per se* only moderately enhanced regeneration of skeletal muscle (Figures 3B–E and Supplementary Figure 2). In summary, these data suggest that stimulation of non-canonical NF-κB signaling via the LTβR agonist results in impaired regeneration of skeletal muscle in wt mice while inhibition of non-canonical NF-κB signaling does not affect the regeneration process.

Interestingly though, numbers of self-renewing MuSCs (Pax7^+^/MyoD^−^) were not altered in both conditions while the number of activated MuSCs (Pax7^+^/MyoD^+^) and further differentiated cells (Pax7^−^/MyoD^+^) was reduced after stimulation of non-canonical NF-κB signaling with the LTβR agonist (Figures 3F–H). This further supports our notion that stimulation of non-canonical NF-κB signaling impairs myogenic differentiation and interferes with MuSC functionality.

**FIGURE 4:**
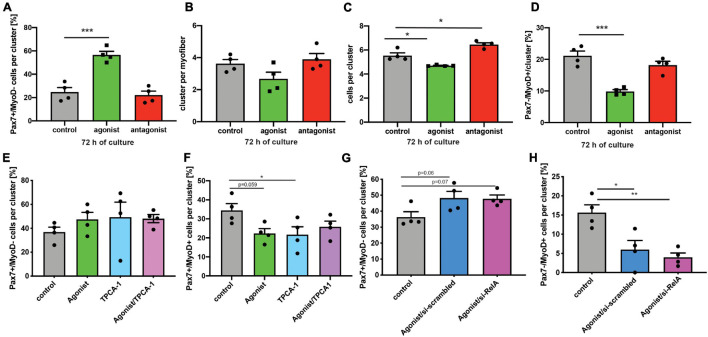
Activation of non-canonical NF-κB signaling with the LTβR agonist impairs differentiation of MuSCs. **(A)** Culture of isolated myofibers for 72 h in the presence of the LTβR agonist results in an increase in the percentage of Pax7^+^/MyoD^−^ cells per cluster while addition of the LTβR antagonist has no effect. **(B)** Culture of isolated myofibers for 72 h in the presence of the LTβR agonist or the antagonist does not affect cluster number. **(C)** Culture of isolated myofibers for 72 h in the presence of the LTβR agonist leads to a reduction in the number of cells per cluster while culture in the presence of the antagonist increases the number of cells per cluster. **(D)** Culture of isolated myofibers for 72 h in the presence of the LTβR agonist results in a decrease in the percentage of Pax7^−^/MyoD^+^ cells per cluster while addition of the LTβR antagonist has no effect. **(E)** Culture of isolated myofibers for 72 h in the presence of the LTβR agonist and/or the IKKB inhibitor results in an increase in the percentage of Pax7^+^/MyoD^−^ cells per cluster. **(F)** Culture of isolated myofibers for 72 h in the presence of the LTβR agonist and/or the IKKB inhibitor results in a reduction in the percentage of Pax7^+^/MyoD^+^ cells per cluster. **(G)** Culture of isolated myofibers for 72 h in the presence of the LTβR agonist results in an increase in the percentage of Pax7^+^/MyoD^−^ cells per cluster independent of knockdown of *relA*. **(H)** Culture of isolated myofibers for 72 h in the presence of the LTβR agonist results in a reduction in the percentage of Pax7^+^/MyoD^+^ cells per cluster independent of knockdown of RelA. *n* = 4 C57BL/6, 3 months of age. *p < 0.05, **p < 0.01, ***p < 0.001. Error bars represent SEM.

Next, we asked whether stimulation of non-canonical NF-κB signaling is directly affecting MuSCs or if the effect on regeneration of skeletal muscle is mediated by other cells. Therefore, we performed experiments using the floating myofiber culture system. Here, MuSCs are cultured on their adjacent myofibers, which allows their direct manipulation while being cultured in their endogenous niche (Hüttner et al., 2019). After 72 h of culture single MuSCs form clusters of cells on the myofiber which can then be examined for their differentiation status, e.g., by immunostaining for Pax7 and MyoD (a marker for early differentiation). Stimulation of non-canonical NF-κB signaling by the LTβR agonist led to an increase in the percentage of Pax7^+^/MyoD^−^ cells, which are non-differentiated, self-renewing cells. Interestingly, inhibition of the NF-κB signaling by the LTβR antagonist also did not affect the percentage of Pax7^+^/MyoD^−^ cells (Figure 4A). However, stimulation of NF-κB signaling by the LTβR agonist did not cause a significant change in the number of clusters per myofiber (Figure 4B) while the number of cells per cluster and the amount of differentiating MuSCs (Pax7^−^/MyoD^+^) were reduced (Figures 4C,D). Of note, also here inhibition of NF-κB signaling via the LTβR antagonist had no effect. To conclude that indeed differentiation of MuSCs is impaired by activation of NF-κB signaling with the LTβR agonist, we investigated different time points (Supplementary Figure 3). Thereby we show that the percentage of non-differentiated MuSCs (Pax7^+^/MyoD^−^) was already increased after 24 h and 48 h of culture while the total number of MuSCs per myofiber was not affected (Supplementary Figures 3A–D). Also at these early time points, inhibition of NF-κB signaling by the LTβR antagonist did not affect MuSC behavior. A closer look at the clusters formed after 72 h of culture in the presence of the LTβR agonist confirmed our observation that activation of NF-κB signaling impairs myogenic differentiation, e.g., demonstrated by the reduced percentage of Pax7^+^/MyoD^+^ MuSCs being further differentiated myogenic cells (Supplementary Figures 3E,F).

To get a better understanding of the interplay between canonical and non-canonical NF-κB signaling in MuSCs, we cultured MuSCs on floating myofibers in the presence of the LTβR agonist and inhibitors of canonical NF-κB signaling (Figures 4E–H and Supplementary Figure 4). Interestingly, we observed very similar effects on myogenic differentiation of MuSCs when they were cultured in the presence of the LTβR agonist alone or if canonical NF-κB signaling was inhibited in addition. Of note, we observed similar effects when using TPCA-1, IMD0354 and siRNA targeting RelA in addition to the LTβR agonist. However, the effects on MuSC differentiation we observed with TPCA-1 might be caused by inhibition of JAK/STAT signaling since this inhibitor was described to also affect signaling via STAT3, a pathway known to be important for MuSC functionality (Nan et al., 2014; Price et al., 2014; Tierney et al., 2014). Also IMD0354 was shown to affect STAT signaling, namely signaling via STAT1 (Tanaka et al., 2005). In all cases we observed an impaired myogenic differentiation suggesting that inhibition of canonical NF-κB signaling cannot compensate for activation of non-canonical NF-κB signaling in MuSCs.

### Inhibition of Non-canonical NF-κB Signaling Improves Regeneration in mdx Mice

**FIGURE 5:**
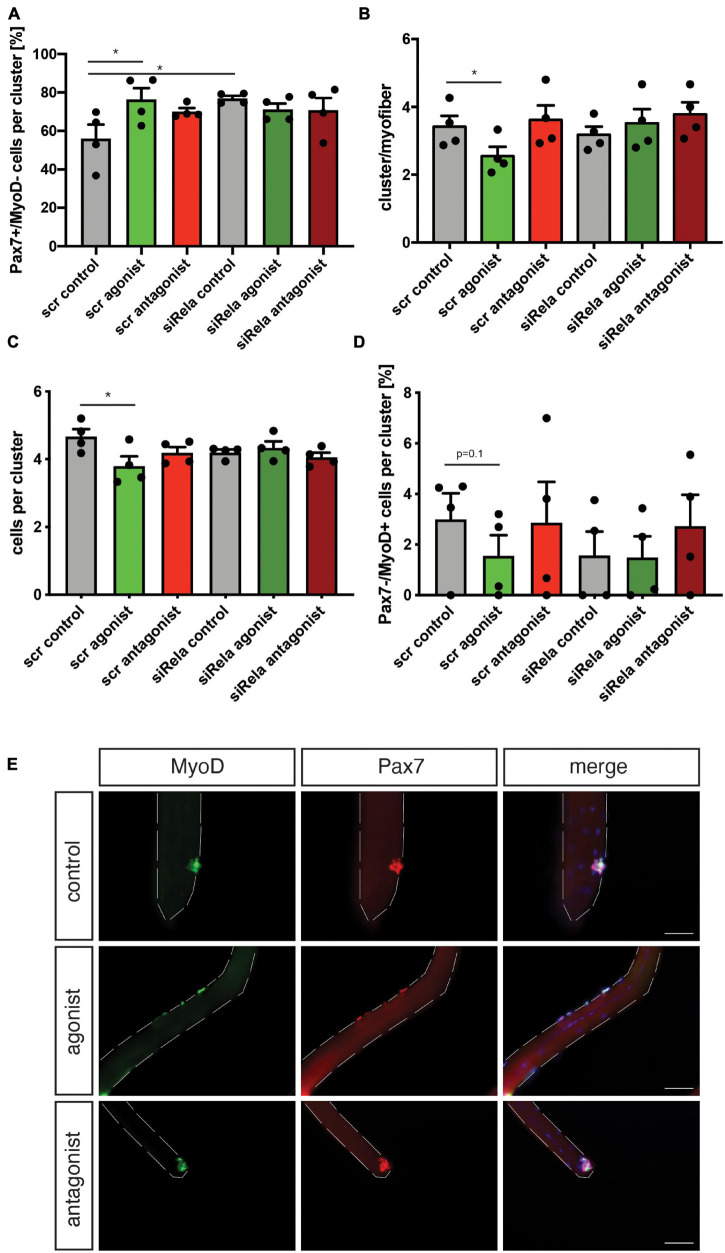
Manipulating the activation of the non-canonical NF-κB signaling pathway affects myogenic differentiation of MuSCs from mdx mice. **(A)** Culture of isolated myofibers for 72 h in the presence of the LTβR agonist causes an increase in the percentage of Pax7^+^/MyoD^−^ independent of knockdown of *relA*. **(B)** Culture of isolated myofibers for 72 h in the presence of the LTβR agonist leads to a reduction in the number of clusters per myofiber independent of knockdown of *relA*. Incubation with the LTβR antagonist does not affect the number of clusters per myofiber. **(C)** Culture of isolated myofibers for 72 h in the presence of the LTβR agonist causes a reduction in the number of cells per cluster while incubation with the LTβR antagonist does not affect the number of cells per cluster. **(D)** Culture of isolated myofibers for 72 h in the presence of the LTβR agonist causes a reduction in the percentage of Pax7^−^/MyoD^+^ independent of knockdown of *relA*. **(E)** Representative images of clusters of MuSCs after culture for 72 h in the presence of the LTβR agonist, LTβR antagonist, or a solvent control. MyoD in green, Pax7 in red. Nuclei are counterstained with DAPI (blue). Scale bar = 50 μm. *n* = 4, 3 months of age, *p < 0.05. Error bars represent SEM.

Next, we asked if stimulation of non-canonical NF-κB signaling with the LTβR agonist impairs MuSC function and thereby regeneration of skeletal muscle in muscles which are continuously regenerating. Therefore, we investigated how stimulation or inhibition of non-canonical NF-κB signaling affects MuSCs from mdx mice, a mouse model for Duchenne muscular dystrophy, often used as a model for continuous muscle regeneration (Dangain and Vrbova, 1984; Anderson et al., 1987; Figure 5A). However, it is known that MuSC divisions in mdx mice are reduced after 72 h of culture compared to wt mice (Dumont et al., 2015). When we stimulated non-canonical NF-κB signaling with the LTβR agonist in MuSCs from mdx mice, we observed a slight reduction in the cluster number per myofiber (Figures 5B,E). This is different from our observation in wt mice, probably due to the different activation status of MuSCs from mdx mice compared to wt mice and their impaired ability to undergo asymmetric divisions (Dumont et al., 2015). Interestingly, inhibition of non-canonical NF-κB signaling did not affect cluster number or composition of clusters in MuSCs from mdx mice (Figures 5B–E). However, the number of Pax7^+^/MyoD^+^ early differentiating cells was reduced after stimulation of non-canonical NF-κB signaling with the LTβR agonist (Figures 5C,E) similar to the effect we observed in wt mice. This reduction in committed cells coincided with a reduction in the number of Pax7^−^/MyoD^+^ cells, which represent further differentiated cells (Figures 5D,E). Also here, the results are in line with the results obtained in wt mice (Figures 4A–D). Of note, neither the number of cells per cluster, the number of self-renewing cells (Pax7^+^/MyoD^−^ cells) nor the number of Pax7^+^/MyoD^+^ cells were significantly changed following manipulation of non-canonical NF-κB signaling in MuSCs from mdx mice (Figure 5). In summary, these data suggest that stimulation of non-canonical NF-κB signaling results in impaired myogenic commitment and early differentiation after acute activation in wt cells (Figure 4) as well as in already activated MuSCs from mice which undergo continuous regeneration (Figure 5). Furthermore, we show that stimulation of non-canonical NF-κB signaling with the LTβR agonist can directly affect MuSCs.

**FIGURE 6:**
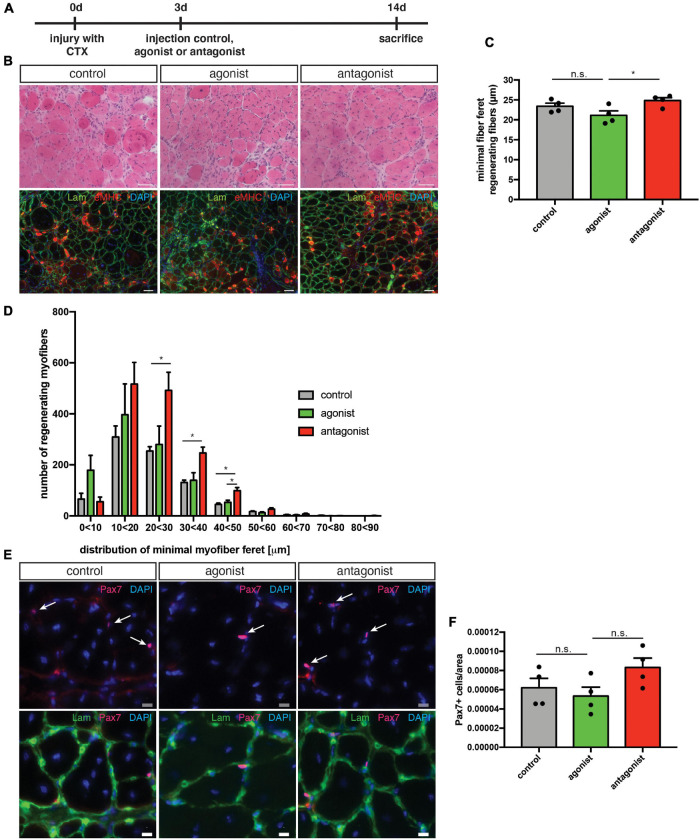
Inhibition of non-canonical NF-κB signaling with the LTβR antagonist improves the regenerative outcome in mdx mice. **(A)** Schematic depicting the experimental setup for CTX injury followed by the injection of the LTβR agonist, LTβR antagonist, or a solvent control. **(B)** Representative images of HE staining (upper panel) and immunostaining for laminin (green) and embryonic MHC (red) (lower panel). Nuclei are counterstained with DAPI (blue). Scale bar: 50 μm. **(C)** Injection of the LTβR antagonist into regenerating muscles results in significantly larger myofibers (measured as minimal fiber feret) compared to muscles injected with the LTβR agonist. **(D)** Myofiber feret distribution of **(C)**. **(E)** Representative images of immunostaining of sections from the respective TA muscles with antibodies directed against laminin (green) and Pax7 (red). Nuclei are counterstained with DAPI (blue) white arrows mark Pax7 positive cells. Scale bar: 10 μm. **(F)** Injection of the LTβR antagonist into regenerating TA muscles from mdx mice results in an increased number of Pax7 positive cells when compared to muscles injected with the LTβR agonist. *n* = 4, 3 months of age, *p < 0.05, ns, not significant. Error bars represent SEM.

Next, we asked whether interfering with the activity of non-canonical NF-κB signaling in muscles undergoing continuous regeneration affects MuSC function and regeneration after acute injury. Therefore, we performed an acute muscle injury of mdx mice followed by intramuscular injection of the LTβR agonist or LTβR antagonist (Figure 6A). We found that stimulation of non-canonical NF-κB signaling only slightly impaired regeneration in mdx mice similar to the effect observed in wt mice (Figures 6B–D). However, inhibition of non-canonical NF-κB signaling resulted in a slight improvement in the regeneration of mdx muscles after acute injury (Figures 6B–D). These results were surprising to us since inhibition of non-canonical NF-κB signaling in wt mice did not affect regeneration (Figure 3). However, dystrophic muscles are characterized by an increased/constant inflammatory response (Manning and O’Malley, 2015). Here, IκB kinase/NF-κB (IKK/NF-κB) signaling was shown to be persistently elevated in immune cells and regenerating myofibers (Acharyya et al., 2007). One might therefore speculate that the improved regeneration in mdx mice after inhibition of non-canonical NF-κB signaling is not due to a direct effect on MuSCs but rather an indirect one mediated by the immune cells. Next, we quantified MuSC numbers in mdx mice after stimulation or inhibition of non-canonical NF-κB signaling. Here, stimulation of non-canonical NF-κB signaling did not affect MuSC numbers while its inhibition resulted in a slight increase, reminiscent of the effect observed in wt mice (Figures 6E,F). In summary, these results suggest that stimulation of non-canonical NF-κB signaling via the LTβR agonist impairs myogenic differentiation and regeneration of muscles after acute injury or undergoing continuous regeneration. However, inhibition of non-canonical NF-κB signaling improves regeneration of muscles of mdx mice. We hypothesize that inhibition of non-canonical NF-κB signaling primarily affects non-myogenic cells in mdx muscles and that improvements of regeneration are rather secondary effects in this specific mouse model.

### Stimulation of Non-canonical NF-κB Signaling Impairs Myogenic Differentiation Independent of Dystrophin Status in Human Myoblasts

**FIGURE 7:**
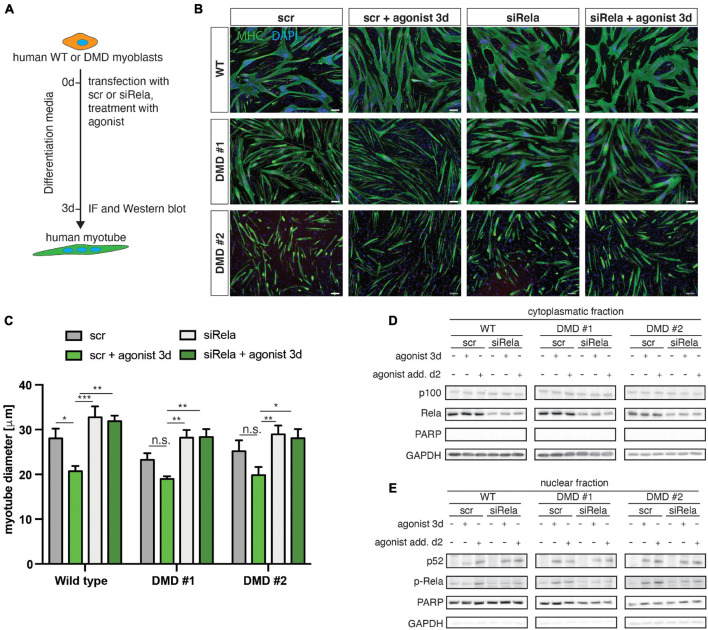
Activation of non-canonical NF-κB signaling by the LTβR agonist impairs myogenic differentiation in human myoblasts from healthy donors and dystrophic patients. **(A)** Experimental scheme of myoblast differentiation in the presence of the LTβR agonist and transfection with a siRNA targeting *rela*. **(B)** Representative images of myotubes stained with antibodies directed against myosin heavy chain (MHC, green) and myogenin (red). Nuclei are counterstained with DAPI (blue). Scale bar = 50 μm. **(C)** Culture in the presence of the LTβR agonist impairs myogenic differentiation in human primary myoblasts isolated from healthy control or from DMD patients. siRNA mediated knockdown of *RELA* results in increased myotube diameters in culture conditions with and without the LTβR agonist. **(D)** Culture in the presence of the LTβR agonist for 3 days or 24 h causes cleavage of p100 to active p52 and increased phosphorylation of RelA **(E)**. siRNA mediated knockdown of *RELA* and culture in the presence of the LTβR agonist results in phosphorylation of RelA and cleavage of p100 to p52. *n* = 3. *p < 0.05, **p < 0.01, ***p < 0.001. Error bars represent SEM.

We next wondered whether loss of dystrophin expression affects the response to stimulation or inhibition of non-canonical NF-κB signaling also in myogenic cells from humans. Therefore, we investigated myogenic differentiation of human myoblasts from DMD patients and healthy control in response to stimulation of non-canonical NF-κB signaling with the LTβR agonist (Figure 7A). Stimulation of non-canonical NF-κB signaling resulted in impaired myogenic differentiation, reminiscent of the phenotype observed in murine myoblasts (Figures 7B,C). Interestingly, we found a very similar response in DMD patient derived myoblasts to stimulation of non-canonical NF-κB signaling (Figures 7B,C). However, we observed enhanced myogenic differentiation after inhibition of canonical NF-κB signaling with a siRNA targeting *RELA*. Of note, knockdown of *RELA* enhanced myogenic differentiation independent of stimulation of non-canonical NF-κB signaling in myoblasts from healthy control and DMD patients (Figures 7B,C and Supplementary Figure 5). In accordance with the data we obtained in murine myotubes we found a slight activation of the canonical NF-κB pathway following stimulation with the LTβR agonist independent of the dystrophin status (Figures 7D,E and Supplementary Figure 5). We conclude that stimulation of non-canonical NF-κB signaling with the LTβR agonist inhibits myogenic differentiation in murine and human myoblasts independent of the dystrophin status.

In summary, we demonstrate that stimulation of non-canonical NF-κB signaling impairs myogenic differentiation. We show that the LTβR agonist directly affects MuSCs and their progeny causing impaired regeneration of skeletal muscle.

## Discussion

Myogenesis and regeneration of skeletal muscle are tightly controlled processes requiring the precise activation and inhibition of signaling pathways to allow proper differentiation and self-renewal (Schmidt et al., 2019; Henze et al., 2020). Canonical and non-canonical NF-κB signaling are important signaling pathways whose tight regulation is required for cellular processes such as differentiation, cell adhesion, migration or inflammation, and survival. Of note, non-canonical NF-κB signaling was shown to be important for the regulation of differentiation processes (Shih et al., 2011; Zhang et al., 2017). However, a crosstalk between canonical and non-canonical NF-κB signaling is thought to occur during differentiation, e.g., during osteogenic differentiation. While Caron et al. (2012) showed that RelA/p65 mediated signaling facilitates chondrogenic differentiation, a study by Franzoso et al. (1997) demonstrated that the simultaneous knockout of the RelA and RelB binding partners p50 and p52 resulted in impaired osteogenic differentiation causing osteopetrosis in mice. However, deletion of p100 – the precursor of p52 – results in endochondral ossification in mice, a phenotype which can be partially rescued by additional deletion of RelB. This further supports the notion that a regulated crosstalk between canonical and non-canonical NF-κB signaling is important for proper differentiation (Jimi et al., 2019). Regarding the role of NF-κB signaling in myogenesis and regeneration of skeletal muscle, research mainly focused on members of the canonical NF-κB signaling axis. For instance, the importance of canonical NF-κB signaling for the control of myogenic differentiation was demonstrated using p65/RelA deficient myoblasts (Bakkar et al., 2008; Bakkar and Guttridge, 2010).

Here, we focused on the role of non-canonical NF-κB signaling in myogenic differentiation, *in vitro* and *in vivo*. Thereby, we demonstrate that stimulation of non-canonical NF-κB signaling through an agonistically acting antibody directed against LTβR (LTβR agonist) impairs myogenic differentiation of myoblasts and MuSCs. We suggest that this is one of the reasons for the impaired regeneration of skeletal muscle following activation of the non-canonical NF-κB signaling pathway (Supplementary Figure 6). Through experiments using the floating myofiber culture system, we demonstrate that activation of the non-canonical NF-κB signaling pathway can also directly affect myogenic differentiation. We show that non-canonical NF-κB signaling regulates myogenic differentiation and that overly active non-canonical NF-κB signaling is detrimental for progression of myogenic differentiation and thereby regeneration of skeletal muscle. While stimulation of non-canonical NF-κB signaling impairs myogenic differentiation in wt mice, inhibition of non-canonical NF-κB signaling with an antagonistically working antibody directed against the LTβR (LTβR antagonist) does not affect myogenic differentiation or MuSC function, neither *in vivo* nor *in vitro*. However, inhibition of non-canonical NF-κB signaling in mdx mice during acute regeneration improves the regenerative outcome (Figure 6). We suggest that the enhanced regeneration in mdx mice is caused by secondary effects of the LTβR antagonist, e.g., by blocking the elevated inflammatory response of invading immune cells observed in dystrophic muscles and not by direct effects on MuSCs (Manning and O’Malley, 2015). This hypothesis is supported by the finding that differentiation of MuSCs on floating myofibers from mdx mice is not improved by manipulating the activity of non-canonical NF-κB signaling. Furthermore, differentiation of myoblasts derived from healthy controls or DMD patients is similarly impaired after stimulation of non-canonical NF-κB signaling suggesting that overly active non-canonical NF-κB signaling inhibits myogenic differentiation in a similar manner in mice and men. Furthermore, these data support our notion that stimulation of non-canonical NF-κB signaling directly targets myogenic cells while inhibition of non-canonical NF-κB signaling rather affects non-myogenic cells.

Stimulation of non-canonical NF-κB signaling in myoblasts might lead to impaired activation of the myogenic transcription factor cascade required for proper myogenic differentiation (Hernandez-Hernandez et al., 2017). Increased levels of RelB in the nucleus might interfere with binding of MyoD or myogenin to specific promoter regions thereby impairing myogenic differentiation. However, changes in mRNA stabilization of *myod* or *myogenin* could also cause impaired differentiation after stimulation with the LTβR agonist, similar to RelA interfering with *myoD* stability (Guttridge et al., 2000). In addition to changes in *myoD* or *myogenin* stability, modulation or interaction with TWEAK might contribute to the impaired regeneration/MuSC function following stimulation of non-canonical NF-κB signaling. TWEAK (TNFα-like weak inducer of apoptosis), a member of the TNF family, was shown to regulate myoblast fusion through non-canonical NF-κB signaling in skeletal muscle (Enwere et al., 2012, 2014). Deregulation of Pax7 expression or function might also contribute to the impaired differentiation of MuSCs following stimulation of non-canonical NF-κB signaling. He et al. (2013) demonstrated that cancer cachexia leads to stimulation of canonical NF-κB signaling in MuSCs which causes increased Pax7 levels. Pax7 was shown to be required for myogenic differentiation and MuSC functionality, e.g., Pax7 deficient mice display severe malformations of skeletal muscle and die a couple of weeks after birth (Seale et al., 2000). In contrast, Straughn et al. (2018) demonstrated that inhibition of specific components of canonical NF-κB signaling causes precocious differentiation of MuSCs. However, we observed impaired myogenic differentiation of MuSCs and myoblasts after stimulation of non-canonical NF-κB signaling suggesting that non-canonical NF-κB signaling limits myogenic differentiation, potentially through increasing Pax7 levels or *myoD* mRNA stability.

Of note, we observed a slight upregulation of members of the canonical NF-κB signaling pathway when we stimulated non-canonical NF-κB signaling with the LTβR agonist (Supplementary Figures 1, 5). This suggests an immediate crosstalk of canonical and non-canonical NF-κB signaling during myogenic differentiation. These findings are in line with data which revealed crosstalk of canonical and non-canonical NF-κB signaling and activation of first RelA/p50 and then RelB/p52 after induction of LTβR in mouse embryonic fibroblasts (Muller and Siebenlist, 2003; Basak et al., 2007). The idea of a direct crosstalk between the NF-κB signaling pathways is further supported by another report, in which a site-activation of the canonical NF-κB pathway during adipocyte differentiation was observed when non-canonical NF-κB signaling was stimulated by the agonistically acting antibody directed against LTβR (Weidemann et al., 2016). Activation of non-canonical NF-κB signaling is generally thought to be related to developmental cues (Shih et al., 2011). However, developmental LTβR signaling causes the disruption of the IKBβ inhibitory complex; this then allows the translocation of RelA/p50 into the nucleus (Basak et al., 2007). This mechanism shown in fibroblasts might also occur in myogenic differentiation since we observed an increase in nuclear RelA protein upon stimulation of the LTβR. This might mediate the crosstalk between canonical and non-canonical NF-κB signaling during myogenesis.

The importance of a fine regulation of canonical and non-canonical NF-κB signaling for proper myogenesis is further underscored by studies in dystrophic mice. Here, canonical NF-κB signaling was shown to be overly active (Acharyya et al., 2007). The phenotype of mdx mice was improved, when the aberrant activation of canonical NF-κB signaling in dystrophic conditions was controlled by orally available inhibitors of p65/RelA (Hammers et al., 2016). Interestingly, the authors also observed a slight decrease in activation of non-canonical NF-κB signaling in mdx muscles. We suggest that crosstalk between canonical and non-canonical NF-κB is occurring during myogenic differentiation and that is important for keeping an adequate balance of myogenic progression and self-renewal.

In summary, our study demonstrates that stimulation of non-canonical NF-κB signaling with an agonistically acting LTβR antibody impairs myogenic differentiation, MuSC function and thereby regeneration of skeletal muscle. Furthermore, we provide evidence that a fine balance in the activation of canonical and non-canonical signaling is required for proper myogenic differentiation and MuSC function.

## Data Availability Statement

The raw data supporting the conclusions of this article will be made available by the authors upon request, without undue reservation.

## Ethics Statement

The animal study was reviewed and approved by the Thüringer Landesamt für Lebensmittelsicherheit und Verbraucherschutz.

## Author Contributions

MS, CP, and JM designed and performed the most experiments, analyzed the data, and interpreted the results. AB generated patient derived myoblasts. MS, AW, and JM analyzed the data, interpreted the results, and wrote the manuscript. All authors contributed to the article and approved the submitted version.

## Conflict of Interest

The authors declare that the research was conducted in the absence of any commercial or financial relationships that could be construed as a potential conflict of interest.

## Publisher’s Note

All claims expressed in this article are solely those of the authors and do not necessarily represent those of their affiliated organizations, or those of the publisher, the editors and the reviewers. Any product that may be evaluated in this article, or claim that may be made by its manufacturer, is not guaranteed or endorsed by the publisher.

## Supplementary Material

Supplementary Figure 1 Canonical and non-canonical NF-κB pathway during myogenesis. Separation of cytoplasmatic **(A)** and nuclear fraction **(B)** from primary murine myoblasts undergoing differentiation shows activation of canonical (p-RelA) and non-canonical (p52) signaling during myogenic differentiation. **(C,D)** Culture in the presence of the LTβR agonist for the respective times causes cleavage of p100 to active p52 and increased phosphorylation of RelA. **(E)** Differentiation of primary myoblasts in the presence of the LTβR agonist results in a reduced myotube diameter while addition of the LTβR antagonist does not affect myotube size. Inhibition of the IKKB with TPCA-1 increases the myotube diameter. **(F)** Differentiation of primary myoblasts in the presence of the LTβR agonist results in a reduced myotube diameter while addition of the LTβR antagonist does not affect myotube size. Knockdown of RelA results in an increase in myotube diameter. Scale bar = 50 μm. *n* = 4, 3 months of age, ^∗^*p* < 0.05; ^∗∗^*p* < 0.01, ^∗∗∗^*p* < 0.001. Error bars represent SEM.

Supplementary Figure 2 CTX mediated injury of TA muscles from wt mice followed by injection of the LTβR agonist or LTβR antagonist. **(A)** TA muscles from mice injected with the LTβR antagonist are slightly heavier than muscles injected with the LTβR agonist. **(B)** Myofiber size distribution of eMHC-positive myofibers, *n* = 5. Error bars represent SEM.

Supplementary Figure 3 Activation of non-canonical NF-κB signaling with the LTβR agonist impairs early myogenic differentiation of MuSCs. **(A)** Culture of isolated myofibers for 24 h in the presence of the LTβR agonist results in an increase in the percentage of Pax7^+^/MyoD^−^ cells per cluster while addition of the LTβR antagonist has no effect. **(B)** Culture of isolated myofibers for 24 h in the presence of the LTβR agonist or antagonist does not affect the number of cells per myofiber. **(C)** Culture of isolated myofibers for 48 h in the presence of the LTβR agonist results in an increase in the percentage of Pax7^+^/MyoD^−^ cells per cluster while addition of the LTβR antagonist has no effect. **(D)** Culture of isolated myofibers for 48 h in the presence of the LTβR agonist or antagonist does not affect the number of cells per myofiber. **(E)** Culture of isolated myofibers for 72 h in the presence of the LTβR results in an increase in the number of single cells per myofiber while the percentage of Pax7^+^/MyoD^+^ cells per cluster is reduced. **(F)** The LTβR antagonist does not affect the number of single cells per myofiber nor the percentage of Pax7^+^/MyoD^+^ cells per cluster. *n* = 4, 3 months of age, ^∗^*p* < 0.05, ^∗∗^*p* < 0.01. Error bars represent SEM.

Supplementary Figure 4 MuSC differentiation is impaired after activation of non-canonical NF-κB signaling with the LTβR agonist independent of inhibition of the canonical NF-κB pathway. **(A)** Culture of MuSCs on their adjacent myofibers for 72 h in the presence of the LTβR agonist and an inhibitor of IKKB results in a reduction in the number of cells per cluster. **(B)** The percentage of Pax7^−^/MyoD^+^ cells cell per cluster after 72 h in culture under the respective conditions. *n* = 4, 3 months of age, ^∗^*p* < 0.05; ^∗∗^*p* < 0.01, ^∗∗∗^*p* < 0.001. Error bars represent SEM.

Supplementary Figure 5 Activation of canonical and non-canonical NF-κB signaling in differentiating human myoblasts. Investigation of activation of canonical (RelA, p-RelA) and non-canonical (p100, p52, and LTβR) NF-κB total protein levels by immunoblot analyses. Incubation with the LTβR agonist results in increased phosphorylation of RelA and also cleavage of p100 to p52. Knockdown of *RELA* leads to the activation of the non-canonical pathway after addition of the LTβR agonist, while the phosphorylation status of RelA is not affected.

Supplementary Figure 6 Overly active non-canonical NF-κB signaling impairs myogenic differentiation, muscle stem cell function, and regeneration of skeletal muscle.
