# Supplementary material for: Stimulation of Non-canonical NF-κB Through Lymphotoxin-β-Receptor Impairs Myogenic Differentiation and Regeneration of Skeletal Muscle
Source: Front Cell Dev Biol. 2021 Oct 5;9:721543. doi: 10.3389/fcell.2021.721543 (PMC8523804; doi:10.3389/fcell.2021.721543)
Supplement: Supplementary Figure 1 — Canonical and non-canonical NF-κB pathway during myogenesis. Separation of cytoplasmatic (A) and nuclear fraction (B) from primary murine myoblasts undergoing differentiation shows activation of canonical (p-RelA) and non-canonical (p52) signaling during myogenic differentiation. (C,D) Culture in the presence of the LTβR agonist for the respective times causes cleavage of p100 to active p52 and increased phosphorylation of RelA. (E) Differentiation of primary myoblasts in the presence of the LTβR agonist results in a reduced myotube diameter while addition of the LTβR antagonist does not affect myotube size. Inhibition of the IKKB with TPCA-1 increases the myotube diameter. (F) Differentiation of primary myoblasts in the presence of the LTβR agonist results in a reduced myotube diameter while addition of the LTβR antagonist does not affect myotube size. Knockdown of RelA results in an increase in myotube diameter. Scale bar = 50 μm. n = 4, 3 months of age, ∗p < 0.05; ∗∗p < 0.01, ∗∗∗p < 0.001. Error bars represent SEM. [file Data_Sheet_1.PDF]

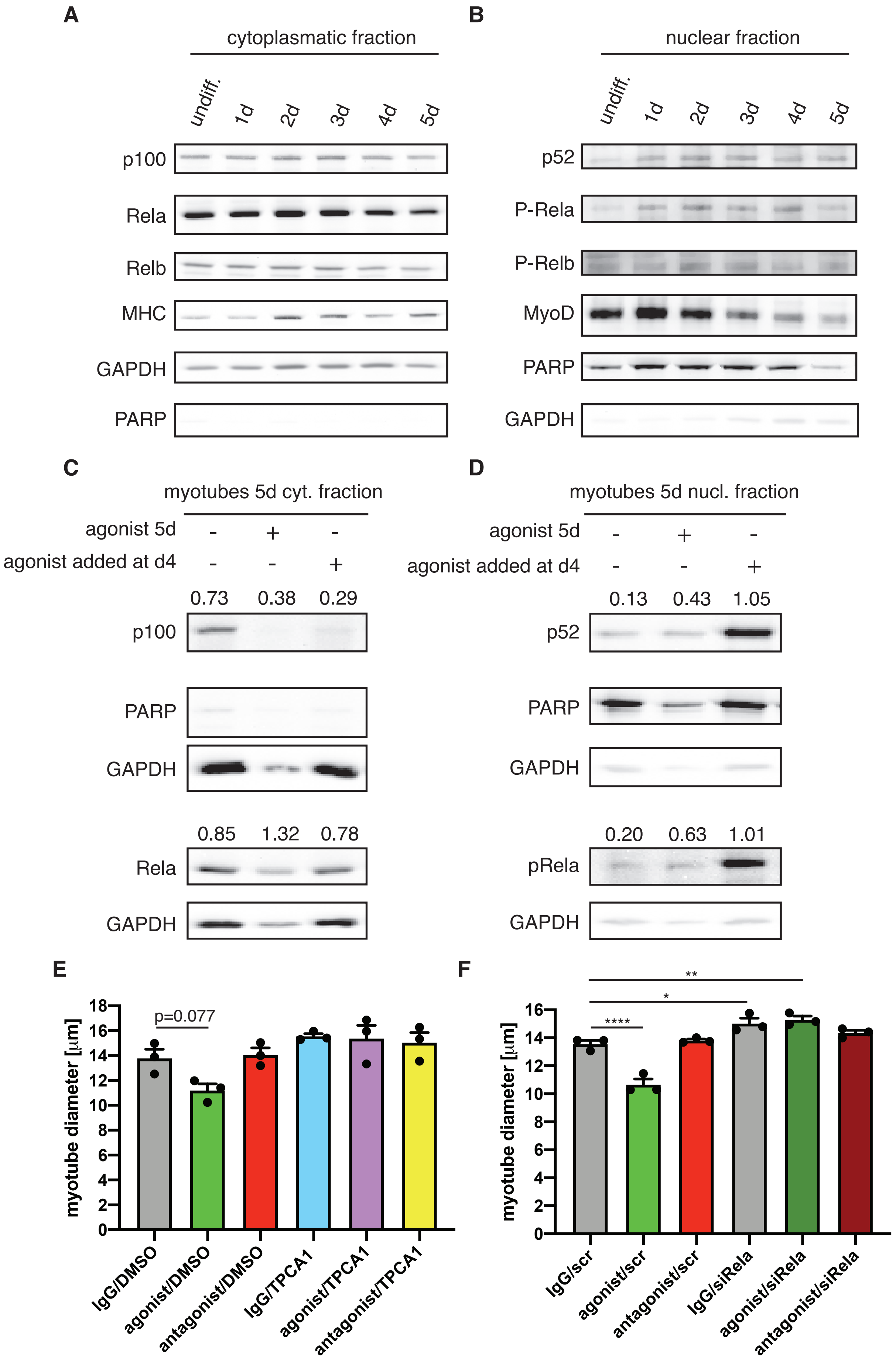
A
cytoplasmatic fraction
undiff.
1d
2d
3d
4d
5d
p100
Rela
Relb
MHC
GAPDH
PARP
B
nuclear fraction
p52
P-Rela
P-Relb
MyoD
PARP
GAPDH
C
myotubes 5d cyt. fraction
agonist 5d
agonist added at d4
0.73 0.38 0.29
p100
PARP
GAPDH
0.85 1.32 0.78
Rela
GAPDH
D
myotubes 5d nucl. fraction
agonist 5d
agonist added at d4
0.13 0.43 1.05
p52
PARP
GAPDH
0.20 0.63 1.01
pRela
GAPDH
E
myotube diameter [µm]
p=0.077
IgG/DMSO
agonist/DMSO
antagonist/DMSO
IgG/TPCA1
agonist/TPCA1
antagonist/TPCA1
F
myotube diameter [µm]
IgG/scr
agonist/scr
antagonist/scr
IgG/siRela
agonist/siRela
antagonist/siRela

Fig.S1

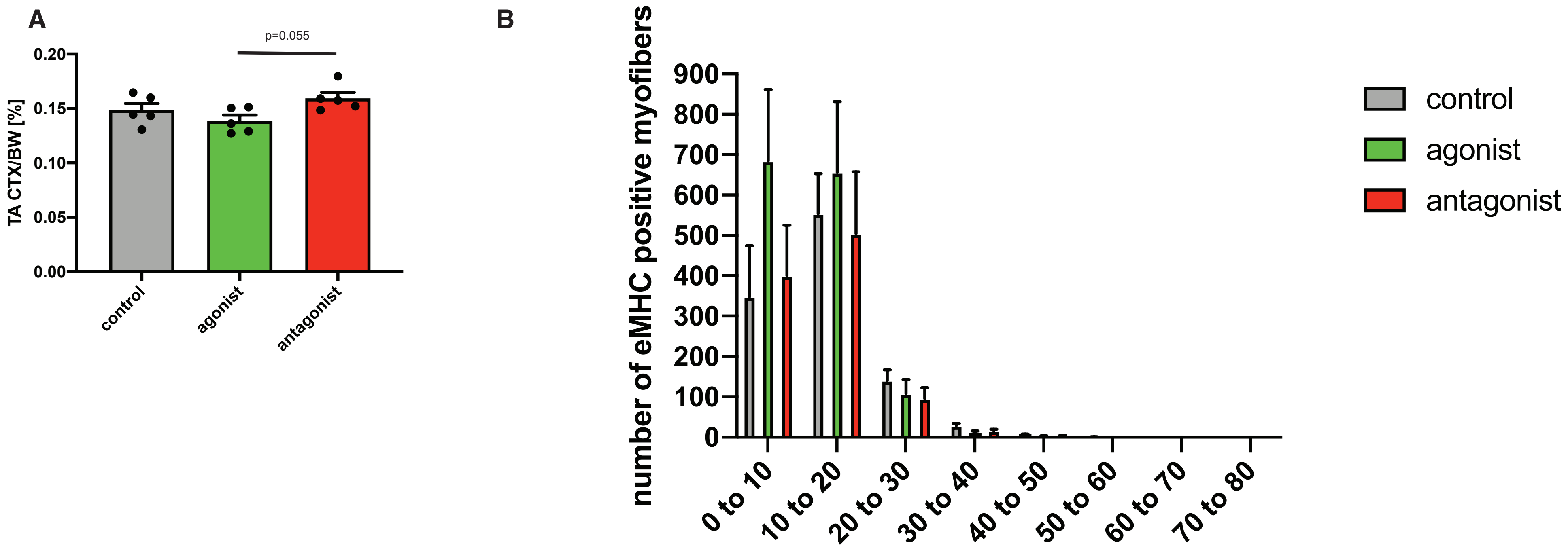
A
p=0.055
TA CTX/BW [%]
0.20
0.15
0.10
0.05
0.00
control
agonist
antagonist
B
number of eMHC positive myofibers
900
800
700
600
500
400
300
200
100
0
0 to 10
10 to 20
20 to 30
30 to 40
40 to 50
50 to 60
60 to 70
70 to 80
control
agonist
antagonist

Fig.S2

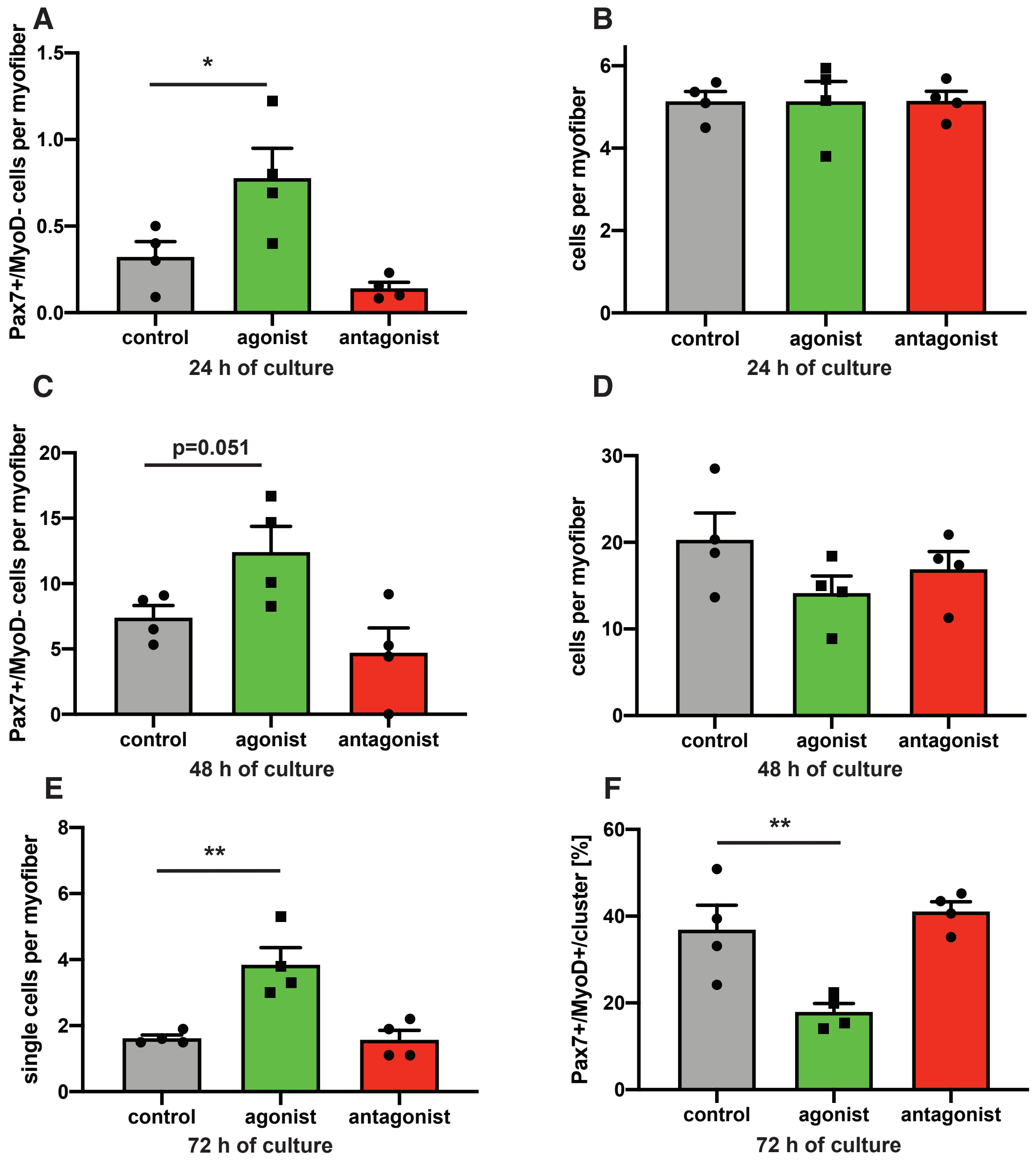
A
Pax7+/MyoD- cells per myofiber
1.5
1.0
0.5
0.0
*
control
agonist
antagonist
24 h of culture
B
cells per myofiber
6
4
2
0
control
agonist
antagonist
24 h of culture
C
Pax7+/MyoD- cells per myofiber
20
15
10
5
0
p=0.051
control
agonist
antagonist
48 h of culture
D
cells per myofiber
30
20
10
0
control
agonist
antagonist
48 h of culture
E
single cells per myofiber
8
6
4
2
0
**
control
agonist
antagonist
72 h of culture
F
Pax7+/MyoD+/cluster [%]
60
40
20
0
**
control
agonist
antagonist
72 h of culture

Fig.S3

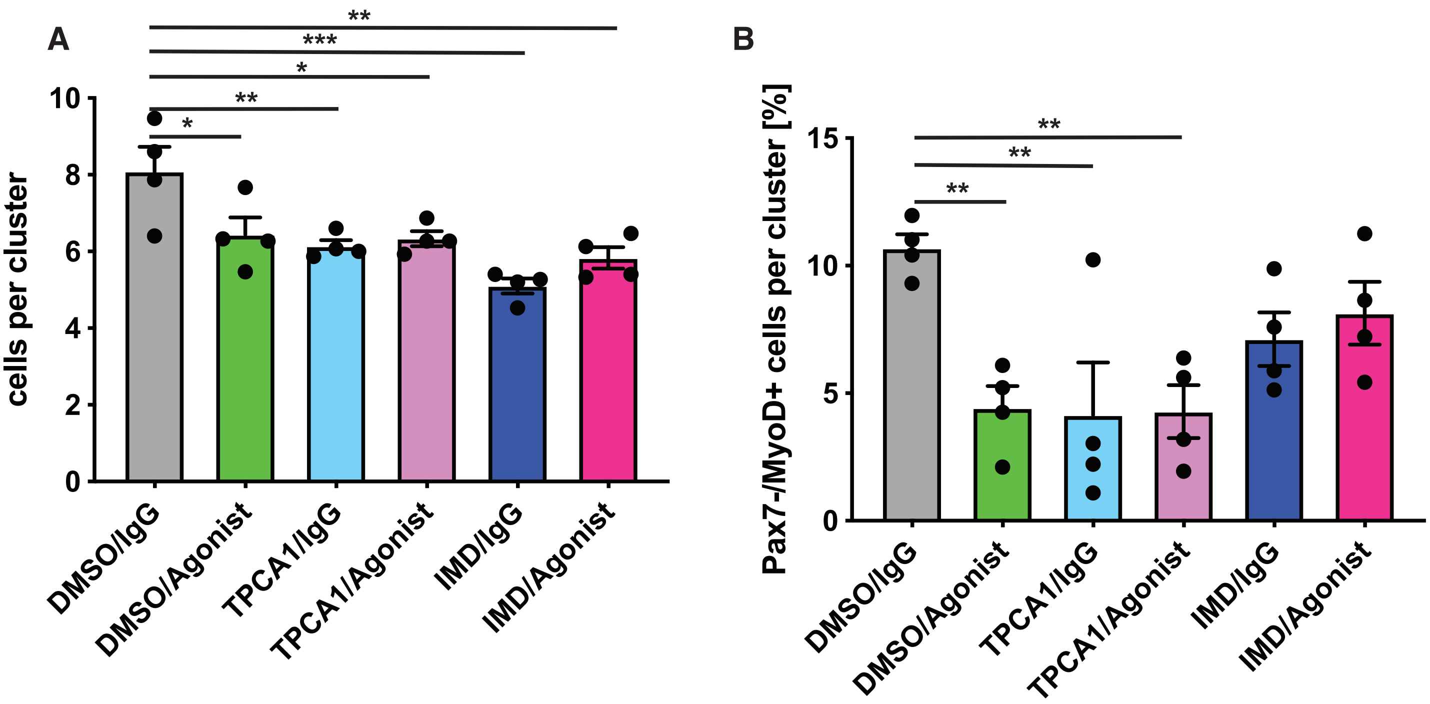
A
B
cells per cluster
Pax7-/MyoD+ cells per cluster [%]
DMSO/IgG
DMSO/Agonist
TPCA1/IgG
TPCA1/Agonist
IMD/IgG
IMD/Agonist

Fig.S4

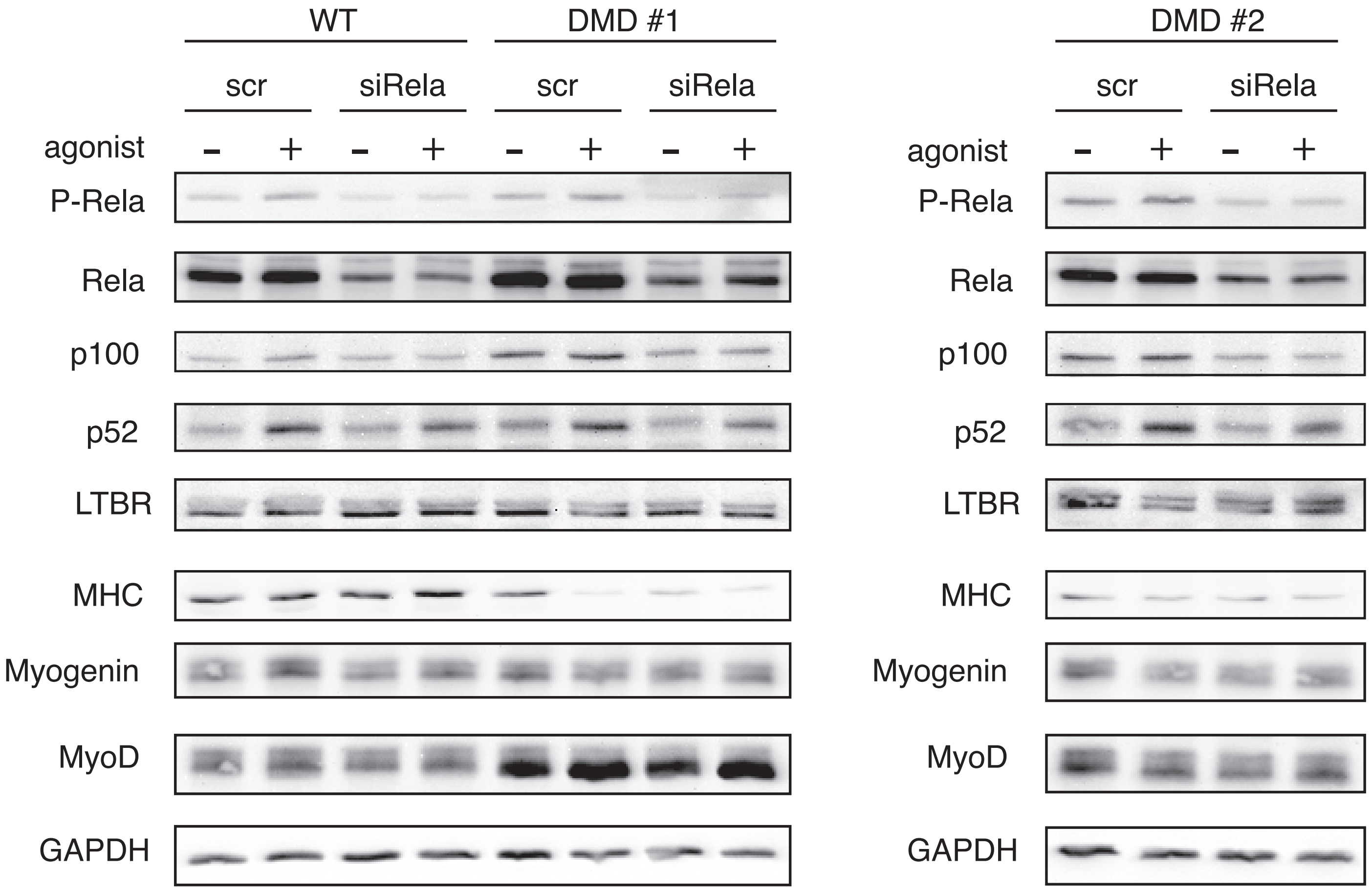
WT
DMD #1
DMD #2
scr
siRela
scr
siRela
scr
siRela
agonist
- + - + - + - +
- + - +
P-Rela
Rela
p100
p52
LTBR
MHC
Myogenin
MyoD
GAPDH

Fig.S5

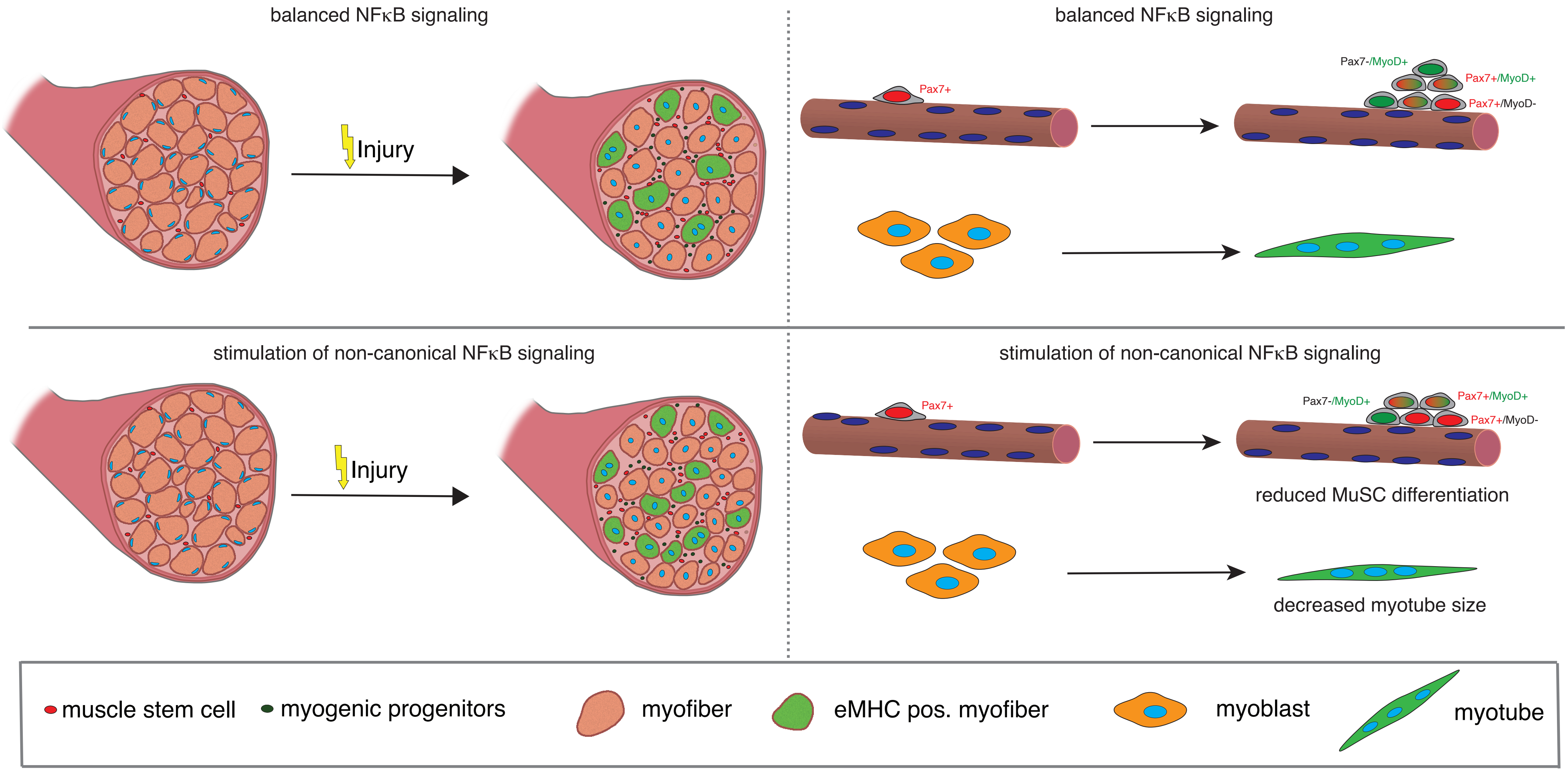

balanced NFκB signaling
Injury
balanced NFκB signaling
Pax7+
Pax7-/MyoD+
Pax7+/MyoD+
Pax7+/MyoD-
stimulation of non-canonical NFκB signaling
Injury
stimulation of non-canonical NFκB signaling
Pax7+
Pax7-/MyoD+
Pax7+/MyoD+
Pax7+/MyoD-
reduced MuSC differentiation
decreased myotube size
muscle stem cell
myogenic progenitors
myofiber
eMHC pos. myofiber
myoblast
myotube
